# Urgent needs in fostering neglected tropical diseases (NTDs) laboratory capacity in WHO Western Pacific Region: results from the external quality assessment on NTDs diagnosis in 2012–2015

**DOI:** 10.1186/s40249-017-0319-x

**Published:** 2017-06-08

**Authors:** Yan Lu, Glenda Gonzales, Shao-Hong Chen, Hao Li, Yu-Chun Cai, Yan-Hong Chu, Lin Ai, Mu-Xin Chen, Hai-Ning Chen, Jia-Xu Chen

**Affiliations:** 1 Key Laboratory of Parasite and Vector Biology, Ministry of Health; WHO Collaborating Center for Tropical Diseases, 207 Ruijin Er Rd, Shanghai, 200025 People’s Republic of China; 2 Malaria, Other Vector borne and Parasitic Diseases (MVP) unit, the World Health Organization Regional Office for the Western Pacific, P.O. Box 2932, 1000 Manila, Philippines; 3 Postal address: National Institute of Parasitic Diseases, Chinese Center for Diseases Control and Prevention, 207 Ruijin Er Rd, Shanghai, 200025 China

**Keywords:** Neglected tropical diseases, Diagnosis, External quality assessment, Western Pacific Region

## Abstract

**Background:**

Neglected tropical diseases (NTDs) are a heterogeneous group of mainly chronic, debilitating and often stigmatizing diseases that largely affects low-income and politically marginalized populations, causing a large burden of public health, social and economies in the NTDs endemic countries. NTDs are caused by infections with a range of pathogen, including bacteria, parasites, protozoa and viruses. The accurate diagnosis of NTDs is important for reducing morbidity, preventing mortality and for monitoring of control programs. External Quality Assessment (EQA), a component of laboratory quality assurance, aims to assess the performance of participating laboratories in detecting parasitic infections. The aim of this paper is to report the findings and put forward the recommendations on capacity build from the EQA results of participating NTDs laboratories in selected countries in the WHO Western Pacific Region from 2012 to 2015.

**Methods:**

Reference or public health laboratories at national level working on NTDs in 6 countries participated in EQAs organized by the National Institute of Parasitic Diseases (NIPD) of Chinese Center for Disease Control and Prevention (CDC) based in Shanghai, China. Two representatives of each participating laboratory were invited to NIPD to detect NTDs’ parasitic infections using the same prepared samples for serological tests (IHA and ELISA) and helminth eggs’ morphological tests (Direct smear and Kato-Katz). All of the results were scored and analyzed by using SPSS statistics 19.0 software.

**Results:**

The percentage of participants who had EQA score ≥ 60 during 2012–2015 for direct smear test were 80.00% (2012), 71.43% (2013), 100% (2014) and 75.00% (2015), whereas for Kato-Katz test were 80.00% (2012), 57.14% (2013), 100% (2014) and 37.50% (2015), respectively. The detection rate of helminth eggs varied in different species, with *Ascaris lumbricoides* being the highest at 94.07% in average. All laboratories did very well with ELISA tests as shown by the high scores in all four years except Lab A in the first and last EQA. For the positive or negative judgments of serum samples, the total coincidence rates of ELISA between 2012 and 2015 were 90.00%, 99.29%, 94.29% and 98.75%, respectively. While the total coincidence rates of IHA were respectively 100%, 95.00%, 90.00% and 97.50%. However, detecting low levels of serum antibody remained problematic for IHA when the titres of samples were taken into consideration.

**Conclusion:**

This study demonstrate that EQA scheme have been beneficial to the participating laboratories. The EQA programme identifies certain deficiencies which were needed to overcome and improved the laboratories’ performance in helminthiasis diagnosis. However, further optimization of accuracy and uniformity in NTDs diagnosis remains a big challenge.

**Electronic supplementary material:**

The online version of this article (doi:10.1186/s40249-017-0319-x) contains supplementary material, which is available to authorized users.

## Multilingual abstracts

Please see Additional file 1 translations of the abstract into the five official working languages of the United Nations.

## Background

Neglected tropical diseases (NTDs) are a heterogeneous group of mainly chronic, debilitating and often stigmatizing diseases that largely affects low-income and politically marginalized people living in rural and urban areas of tropical and subtropical countries, especially in developing regions of sub-Saharan Africa, Asia, and Latin America [1–3].

Currently, there are 17 NTDs prioritized by the World Health Organization (WHO) [1]. These NTDs are endemic in 149 countries and affect an estimated 1.4 billion people. NTDs constitute a very significant burden on the already strained public health systems, social and the economies of many developing countries, therefore may trap people in a vicious cycle of poverty and disease [4, 5]. These diseases are also associated with disfigurement or other sequelae of long-term illness, negative effects on the course and outcome of pregnancy, delayed physical and intellectual development during childhood and reduced productive capacity in older age [2].

The causative agents of NTDs (henceforth NTD pathogens) represent a wide phylogenetic sampling of parasites, protozoa, viruses and bacteria. The main parasitic NTDs include cystiercosis, dracunculiasis, echinococcosis, foodborne trematodiasis (i.e. clonorchiasis, fasciolasis, intestinal fluke infections, ophisthorciasis, paragonimiasis), human African trypanosomiasis, leishmaniasis, lymphatic filariasis, onchocerciasis, schistosomiasis and soil-transmitted helminthiasis (STH, i.e. ascariasis, hookworm infection, strongyloidiasis, trichuriasis) [3, 6, 7].

The NTDs still top the list of health crises in the developing countries. In fact, the burden of infectious diseases in Asia Pacific is second only to that of sub-Saharan Africa [8]. The World Health Organization Regional Office for the Western Pacific (WHO-WPRO) groups together 37 countries and areas, stretching from China and Mongolia in the North and West, to New Zealand and the Pitcairn Islands in the South and East. The region is home to a range of NTDs, and these helminth NTDs put the highest number of people at risk of infection and cause the highest burden due to NTDs in this region [9].

The reliable, sensitive and practical diagnosis of NTDs is important for adequate patient management and for monitoring of control programs. Detection of eggs or larvae and protozoan cysts or trophozoites in feces (sputum, blood, or urine) is the most commonly used approach for the diagnosis of intestinal helminthes and protozoan infections. Several parasitological diagnostic methods are available, such as direct smear method, Kato-Katz thick smears method, sedimentation techniques, the formalin-ethyl-acetate technique, and FLOTAC technique, etc. [10]. The Kato-Katz method, based on duplicate slides, has been the gold standard technique for the detection and quantification of STH and intestinal trematode infections globally and is recommended by WHO for the detection of these infections [11]. Immunodiagnosis (indirect diagnosis) with higher sensitivity and ease of use over stool examination is applicable for most of the NTDs. Therefore, different types of immunological tests (indirect hemagglutination assays (IHA), indirect fluorescence antibody test (IFAT), enzyme linked immunosorbent assay (ELISA), immuno-blotting, etc.) are used in the laboratory diagnosis of parasitic infections, and many immunodiagnostic kits are commercially available at present.

Accurate and timely diagnosis of NTDs reduces morbidity, prevents mortality and enables national NTD programmes to have a reliable mapping and surveillance of NTDs to scale up interventions. To achieve continuous accuracy and quality improvement of diagnosis, external quality assessment (EQA) is commonly used by laboratories, which an EQA provider distributes blinded panels to laboratories for detection and then analyses and reports the results. Access to quality diagnosis is also one of the critical components of the *Regional Action Plan for Neglected Tropical Diseases in the Western Pacific Region (2012–2016)* [12]. Thus, the National Institute of Parasitic Diseases (NIPD) of China CDC based in Shanghai, China, a WHO Collaborating Centre for Tropical Diseases, has initiated an annual regional EQA of national reference or public health laboratories in detecting NTDs’ parasitic infections in 2012. The EQA is funded by WHO and the goal is to assess the performance of the participating laboratories in detecting parasitic infections. This article reports the findings and put forward the recommendations on capacity build from the EQA results of NTDs laboratories in the WHO Western Pacific Region from 2012 to 2015.

## Methods

### Participating laboratories

Nine laboratories working on NTDs in 6 countries invited for the EQAs were pre-selected by WHO. These are: 1) National Center for Parasitology, Entomology and Malaria Control Program, Cambodia, 2) Center of Malariology, Parasitology and Entomology in Lao PDR, 3) University of Malaya in Kuala Lumpur, Malaysia, 4) Research Institute for Tropical Medicine in the Philippines, 5) National Institutes of Malariology, Parasitology and Entomology, Hanoi, Viet Nam, 6) Institute of Malariology, Parasitology and Entomology Ho Chi Minh City, Viet Nam, 7) Institute of Malariology Parasitology and Entomology Quy Nhon, Viet Nam, 8) Anhui Provincial Institute of Parasitic Diseases, China, 9) Jiangxi Provincial Institute of Parasitic Diseases, China. The criteria for selection was reference or public health laboratories at national level working on NTDs diagnosis.

The EQAs implemented by NIPD had no subscription or enrollment fees. Letters of invitation were sent to national reference or public health laboratories and they signified their participation through email at no cost. Upon signifying participation, laboratories nominated two staffs to travel to NIPD for a 2-day laboratory assessment. In order to have comparable results, participants should be of the same level. Mid-level staffs, with working experience of 5–10 years, were selected to attend the EQAs.

### Samples preparation

The following important parasite eggs in stool were used in the EQA from 2012 to 2015: (a) *Ascaris lumbricoides*, (b) *Clonorchis sinensis*, (c) *Enterobius vermicularis*, (d) *Fasciolopsis buski*, (e) hookworm, (f) *Hymenolepis nana*, (g) *Paragonimus westermani*, (h) *Schistosoma japonicum*, (i) *Spirometra mansoni*, (j) *Taenia* spp., and (k) *Trichuris trichiura*. All eggs were collected by NIPD. For each species, Kato-Katz thick smears were prepared ahead of time, then the Kato-Katz slides and direct smear slides were examined blind by two trained technicans from NIPD before the EQAs. There were two panels of serum, one to test for IHA and another for ELISA. Each panel consisted of 20 serum samples. The panels were prepared using sera of patients with schistosomiasis (positive samples) and sera for controls (negative samples), which were selected from a sera bank of NIPD. The diagnosis of schistosomiasis was made by the Kato-Katz method for schistosome eggs in the feces. Six smears from two consecutive stool samples were examined and the results were recorded as eggs per gram feces (EPG). The EPG of schistosomiasis patients were among 16–500. The titre (1:40, 1:20 and 1:10) of serum samples of patients with schistosomiasis were determined in advance by using a commercial anti-*Schistosoma* antibody IHA kit (Anhui Anji Pharmaceutical Science and Technology Co. Ltd). The normal sera were anonymous samples from healthy people living in Shanghai where no reported of schistosomiasis and were screened by the commercial IHA kit and anti-*S. japonicum* IgG ELISA kit (Shenzhen Combined Biotech Co., Ltd). All experiments were repeated for three times to ensure the reliability of the results. Standard operating procedures (SOPs), forms and other relevant tools used during the assessment were prepared by NIPD.

### Proficiency testing

Staffs from participating laboratories performed serological tests (IHA and ELISA) and helminth eggs’ morphological tests (direct smear and Kato-Katz) according to the SOPs at the laboratories of NIPD on the same day. Ten specimens for Kato-Katz examination were processed and prepared, only microscopic examination of slides was involved in the assessment. Fecal smear specimens were in 10 microcentifuge tubes with fecal suspensions. The IHA test kit (Anhui Anji Pharmaceutical Science and Technology Co. Ltd) and ELISA test kit (Shenzhen Combined Biotech Co., Ltd) were commercial, standardized products.

### Evaluation of the results

The results from each laboratory were scored according to the following criteria. 1) IHA: A full score of 100 including 5 score of each serum was set up. If one sample was misjudged (positive or negative), then a score of 5 was deducted. If only the titre of one sample was misjudged, then a score of 2.5 was deducted. 2) ELISA: A full score of 100 was set up for the reading the results of each serum sample, including 5 score of each serum. If one sample was misjudged (positive or negative), then a score of 5 was deducted. 3) Direct smear or Kato-Katz: The full score was 100 comprising of 10 for each correct slide separately. When the type of eggs was misjudged, then the full score could not be obtained. Cut-off point for passing was set at 60 for the 4 diagnostic methods used in the EQA.

### Data analysis

The data collected were entered into a Microsoft Excel worksheet and analyzed using SPSS statistics 19.0 software package (IBM, USA). Categorical variable was analyzed using Chi-square test or fisher’s exact test with the two-tailed, *P* value <0.05 was considered to a significant difference.

## Results

### Comparison of the results between four EQAs

Same tests were used in all rounds of EQA for NTD laboratories. All the tests results acquired by helminth eggs’ morphological tests and serological tests were scored and analyzed. Table 1 summarizes the scores of the laboratories from the 4 EQAs conducted, the results of each laboratory was reported in an anonymous way.

**Table 1:** Comparison of EQA results of laboratories

	Scores															
	Direct smear				Kato-Katz				ELISA				IHA			
Laboratories	2012	2013	2014	2015	2012	2013	2014	2015	2012	2013	2014	2015	2012	2013	2014	2015
Lab A	50	70	60	/	12.50	50	60	/	50	100	90	/	97.50	77.50	70	/
Lab B	/	50	60	50	/	50	80	50	/	95	90	90	/	72.50	90	70.00
Lab C	100	90	70	90	100	80	80	40	100	100	100	100	92.50	52.50	55	95.00
Lab D	90	100	100	100	62.50	80	70	100	100	100	80	100	82.50	90.00	95	97.50
Lab E	100	80	65	80	100	90	70	40	100	100	100	100	75.00	72.50	80	92.50
Lab F	/	28	70	40	/	30	60	20	/	100	100	100	/	95.00	95	90.00
Lab G	100	70	65	60	100	90	60	30	100	100	100	100	80.00	70.00	90	82.50
Lab H	/	/	/	100	/	/	/	100	/	/	/	100	/	/	/	100
Lab I	/	/	/	100	/	/	/	90	/	/	/	100	/	/	/	97.50

Lab B and Lab F were not able to participate in the 2012 EQA, Lab A was not able to participate in the 2015 EQA, Lab H and Lab I only participated in the 2015 EQA

Lab A scored high in IHA test in the first round of EQA (2012) while failed three other tests. ELISA, direct smear and Kato-Katz test results all improved but IHA declined from 97.5 to 77.5 in the second EQA (2013). They passed the helminth eggs’ morphological tests (direct smear and Kato-Katz) and serological tests (IHA and ELISA) in the third EQA (2014) but were not able to participate in the fourth EQA (2015). Lab B wasn’t able to participate in 2012 and they did not score well in 2013 as they failed the direct smear and Kato-Katz tests. However their scores improved in 2014, passing all 4 tests. But, they failed two tests (direct smear and Kato-Katz) again in 2015. Lab C received a perfect score with ELISA test in all four years but failed in 2 consecutive years (2013 and 2014) in IHA test. They score well in ELISA, IHA and direct smear, but failed in Kato-Katz in 2015. Lab D passed all tests since 2012, they also got perfect scores (direct smear-100, Kato-Katz 100, ELISA-100 and IHA-97.50) in 2015. Lab E consistently scored a perfect score of 100 in ELISA test from 2012 to 2015. Their scores for IHA and direct smear improved in the fourth EQA, whereas their score for Kato-Katz tests continuously decreased. Lab F wasn’t able to participate in the first EQA. Their scores are high in both ELISA and IHA tests, but they only passed the direct smear and Kato-Katz tests in 2014. Lab G passed all tests except the Kato-Katz in the fourth EQA since 2012, they consistently got a perfect score in ELISA test but their scores in direct smear and Kato-Katz consistently decreased yearly. Lab H and Lab I were participating in the EQA for the first time in 2015, they both scored well for all the tests.

### Analysis of the helminth eggs’ morphological tests

The ratio of laboratories that correctly identified all samples in the direct smear test ranged from 60.00% (2012) to 14.29% (2013 and 2014) and 37.50% (2015), while in the Kato-Katz test ranged from 60.00% (2012) to 0 (2013 and 2014) and 25.00% (2015). The percentage of participants who had EQA score ≥ 60 in different years for direct smear test were 80.00% (2012), 71.43% (2013), 100% (2014) and 75.00% (2015), whereas for Kato-Katz test were 80.00% (2012), 57.14% (2013), 100% (2014) and 37.50% (2015), respectively. The overall past rates (score ≥ 60) for Kato-Katz (68.65%) were lower than for direct smear (82.60%). A very low pass rate of laboratories with 37.50% was observed for Kato-Katz test in 2015, and five laboratories failed to pass the test (Table 2).

**Table 2:** Overview of the helminth eggs’ morphological tests

			Score = 100		100 > Score ≥ 60		Score < 60	
Year	Tests	No. of participated labs	No. of labs	Ratio (%)	No. of labs	Ratio (%)	No. of labs	Ratio (%)
2012	Direct smear	5	3	60.00	1	20.00	1	20.00
	Kato-Katz	5	3	60.00	1	20.00	1	20.00
2013	Direct smear	7	1	14.29	4	57.14	2	28.57
	Kato-Katz	7	0	0	4	57.14	3	42.86
2014	Direct smear	7	1	14.29	6	85.71	0	0
	Kato-Katz	7	0	0	7	100	0	0
2015	Direct smear	8	3	37.50	3	37.50	2	25.00
	Kato-Katz	8	2	25.00	1	12.50	5	62.50

In the direct smear test, the detection rate of helminth eggs varied in different species, with *A. lumbricoides* being the highest at 94.07% in average, followed by hookworm (92.59%), *E. vermicularis* (90.74%), *T. trichiura* (84.81%), *S. japonicum* and *Taenia* spp. (81.48% in average), *C. sinensis* (74.07%), *P. westermani* (66.67%), *F. buski* (59.26%) and least was *S. mansoni* (29.63%). More details showed in Table 3.

**Table 3:** Average detection rate of parasite eggs using direct smear method and Kato-Katz method

Direct smear		Kato-Katz	
Species	Average detection rate	Species	Average detection rate
*A. lumbricoides*	94.07% (25.4/27)	*A. lumbricoides*	74.07% (20/27)
*C. sinensis*	74.07% (20/27)	*C. sinensis*	74.07% (20/27)
*E. vermicularis*	90.74% (24.5/27)	*E. vermicularis*	66.67% (18/27)
*F.buski*	59.26% (16/27)	*F. buski*	59.26% (16/27)
Hookworm	92.59% (25/27)	*H. nana*	50.00% (4/8)
*P. westermani*	66.67% (18/27)	*P. westermani*	40.74% (11/27)
*S. japonicum*	81.48% (22/27)	*S. japonicum*	59.26% (16/27)
*S. mansoni*	29.63% (8/27)	*S. mansoni*	13.64% (3/22)
*Taenia spp.*	81.48% (22/27)	*Taenia* spp.	85.19% (23/27)
*T. trichiura*	84.81% (22.9/27)	*T. trichiura*	84.21% (16/19)
		Negative	90.91% (20/22)

In the Kato-Katz test, the average rate of detection was 64.23%, with the highest for negative slide (90.91%) and the lowest for *S. mansoni* (13.64%). The detection rate of *Taenia spp.* was 85.19%, and detection rate of *T. trichiura* was 84.21%, while *A. lumbricoides* and *C. sinensis* were 74.07%, *E. vermicularis* was 66.67%, *F. buski* and *S. japonicum* were 59.26%, *H. nana* was 50%, *P. westermani* was 40.74% and *S. mansoni* was 13.64% (Table 3).

### Analysis of the serological tests

For the positive or negative judgments of serum samples, the total coincidence rates of ELISA between 2012 and 2015 were 90.00%, 99.29%, 94.29% and 98.75%, respectively. While the total coincidence rates of IHA were respectively 100%, 95.00%, 90.00% and 97.50%. The overall coincidence rates of ELISA for positive samples (97.35%) were higher than those for negative samples (94.64%). On the contrary, the overall coincidence rates of IHA for positive samples (94.37%) were lower than those for negative samples (99.40%) (Table 4).

**Table 4:** Overview of the serological tests

Year	Tests	Coincidence rate for positive samples (%)	Coincidence rate for negative samples (%)	Total coincidence rate (%)
2012	ELISA	92.00% (69/75)	84.00% (21/25)	90.00% (90/100)
	IHA	100% (75/75)	100% (25/25)	100% (100/100)
2013	ELISA	99.05% (104/105)	100% (35/35)	99.29% (139/140)
	IHA	93.33% (98/105)	100% (35/35)	95.00% (133/140)
2014	ELISA	97.62% (41/42)	89.29% (25/28)	94.29% (66/70)
	IHA	83.33% (35/42)	100% (28/28)	90.00% (63/70)
2015	ELISA	100% (80/80)	97.50% (78/80)	98.75% (158/160)
	IHA	96.25% (77/80)	98.75% (79/80)	97.50% (156/160)

When the titres of samples were taken into consideration, the results of IHA test showed that the coincidence rates for serum samples with different titres differed significantly between 2012 and 2015(Chi-square test, *P* < 0.001). The overall coincidence rates for serum samples with titre = 1:40 was better than those for serum samples with titre = 1:20 or 1:10, despite a very low ratio was recorded in 2013 (Table 5). Detecting low levels of serum antibody in patients with schistosomiasis remained problematic for IHA as indicated by the low coincidence rates for serum samples with titre = 1:20 or 1:10 (<50%).

**Table 5:** Coincidence rates for serum samples with different titres

Year	Titres	Coincidence rate (%)
2012	1:40	92.00% (23/25)
	1:20	48.00% (12/25)
	1:10	44.00% (11/25)
2013	1:40	34.30% (12/35)
	1:20	40.00% (14/35)
	1:10	51.43% (18/35)
2014	1:40	85.71% (12/14)
	1:10	42.86% (12/28)
2015	1:40	73.44% (47/64)
	1:10	50.00% (8/16)

## Discussion

EQA scheme assesses the standard of laboratory testing and provide the data required as a starting point for improving standards. EQA can be used to compare laboratory performance, reveal potential problems associated with diagnostic kits or operations, provide objective evidence of testing quality, indicate areas in a laboratory requiring improvement and identify training needs [13]. EQA of laboratories also provides a channel of communication and a source of educational material. Previous reports on EQAs for toxoplasmosis serological testing, the diagnosis of dengue infection, the diagnosis of malaria and sleeping sickness, African public health microbiology laboratories or antimicrobial susceptibility testing demonstrate that the assessments improve the overall quality of testing and identify certain functional deficiencies requiring strengthening among the participating laboratories [14–18].

In this paper, we reviewed the results from the EQAs of national public health laboratories in detecting NTDs’ parasitic infections in 6 countries of the WHO Western Pacific Region. The EQA scheme was conducted yearly in NIPD in Shanghai, China and was participated by nine laboratories from 6 countries. The assessment was expected to increase confidence in laboratory results that are used for diagnosis, surveillance as well as research.

The results of direct smear test and Kato-Katz test showed that eggs of *S. mansoni* was much more easily to be misjudged (average detection rates were 29.63% for direct smear method and 13.64% for Kato-Katz method, respectively). There was a problem for some participants in differentiating eggs of *S. mansoni* from those of *Fasciola hepatica*, *F. buski* and *P. westermani*. Since *S. mansoni* infection can be diagnosed by fecal examination, but adults in human infections are rare. This may probably explain why some laboratories lack the relevant experience in identifying the specie of eggs. It also should be noted that species-specific diagnosis based on the egg morphology is difficult for their similar shapes and sizes. Hence, not only did accurate and species-specific diagnosis require highly qualified laboratory personnel and appropriate equipment, but continued EQA schemes was necessary for enhancing and sustaining diagnostic performance [19].

The detection rates of other species of eggs varied with different diagnostic method (direct smear or Kato-Katz). Some laboratories could identify the eggs (such as *S. japonicum* eggs, *P. westermani* eggs) using direct smear method, but none of them could identify the same eggs using Kato-Katz method. The results of direct smear test and Kato-Katz test indicated that the overall capacity of discriminating the helminthes eggs still needs improvement. The laboratories needed more practice to improve the accuracy of detections.

In the serological tests, all laboratories passed the ELISA and IHA tests in all years, except Lab A in 2012 and Lab C in 2013, when the positive or negative judgments of serum samples only were taken into consideration. While for the determination of the titer, the coincidence rates for serum samples declined significantly and a large variability in the IHA results of different titres was found. One reason for the large variability could be that results of IHA were observed with naked eyes, and were subject to inaccuracy of technicians’ subjective judgment and experience. Contamination was another factor which affects the results. We also found out that some participants had no much experience in using IHA and not highly skilled in serological detection methods. The participants also had problems in judging the results obtained from the kits. The reliable identification of parasitic infections required in-depth training, expertise and experience. Therefore, basic immunological theory was needed for all participants.

This EQA of national public health laboratories in detecting NTDs’ parasitic infections in the Western Pacific Region also had some limitations. First, not all of the participating laboratories were equally familiar with the SOPs and some of the laboratories use different methods and procedures in their routine diagnostic work. Second, some laboratories were not able to participate in every round of EQA due to logistics reason. Without the testing results of these laboratories would affect the comparison between different laboratories and years. Third, samples used in helminth eggs’ morphological tests should also be expanded to include common parasites seen in participating countries. It is important that laboratories know as many parasites as they can because of increasing migration and population movement which also increased the risk of getting infections not common in their own country.

EQA results from 2012 to 2015 show that there is an improvement with the laboratories’ capacity in helminthiasis diagnosis although the participants of each round EQA were not the same persons and there were individual differences in their detection capacity. Furthermore, some of the participants still need improvement especially in the direct smear and Kato-Katz methods. Laboratories who participated in the EQA agree that this activity is a useful way to assess the capacity of laboratories, and that this needs to be continued and sustained.

Participation in an Inter Laboratory Comparison (ILC) programme, such as EQA or proficiency testing, is significant for medical laboratories according to ISO 15189 [20]. It allows for participating laboratories assess whether their testing results are comparable with other laboratories testing results. Moreover, when their results are discrepant with expected results, the EQA provider and participating laboratories are supposed to analyze and manage the results of EQA and implement correctives action to improve its performance levels [14].

Future emphasis may focus on capacity building and strengthening of laboratories in the region, including intensive training, establishment of standardized reference laboratories and sustained external evaluation system. NIPD Shanghai and WHO aim to continuously implement the EQA yearly and increase the number of specimens analyzed, expand to other laboratories and countries not currently participating. Furthermore, the same staffs from participating laboratories will be invited for the next EQAs to avoid the individual differences in their detection capacity affecting the results. We hope the governments in countries around WHO Western Pacific Region will pay more attention to these, especially the establishment of a standardizing network of reference laboratories.

## Conclusion

This study found that a continuous monitoring of laboratories by EQAs has been beneficial to the participating laboratories. The EQA programme identified certain deficiencies which were needed to overcome and improved the laboratories’ performance in helminthiasis diagnosis. However, further optimization of accuracy and uniformity in NTDs diagnosis remains a big challenge. Finally, regular training activities on parasite identification should be conducted nationally or regionally to improve and strengthen the technicians’ skills. EQAs could provide opportunities for knowledge sharing and also be expanded online through website.

## Additional file

Additional file 1: Multilingual abstracts in the five official working languages of the United Nations. (PDF 679 kb)
