# Supplementary material for: Urgent needs in fostering neglected tropical diseases (NTDs) laboratory capacity in WHO Western Pacific Region: results from the external quality assessment on NTDs diagnosis in 2012–2015
Source: Infect Dis Poverty. 2017 Jun 8;6:106. doi: 10.1186/s40249-017-0319-x (PMC5463369; doi:10.1186/s40249-017-0319-x)
Supplement: Additional file 1: — Multilingual abstracts in the five official working languages of the United Nations. (PDF 679 kb) [file 40249_2017_319_MOESM1_ESM.pdf]

Translation of the abstract into the six official working languages of the United Nations

الاحتياجات العاجلة في مجال تعزيز القدرات المعملية للأمراض المدارية المهملة في منطقة منظمة الصحة العالمية غرب المحيط الهادئ: نتائج تقييم الجودة الخارجية في تشخيص الامراض المدارية المهملة في الفترة 2012-2015

يان لو، غليندا غونزاليس، شاو-هونغ تشين، هاو لي، يو تشون تساي، يان-هونغ تشو، لين آي، مو-شين تشين، هاي نينغ تشين، جيا شو تشين

الملخص

الخلفية: الامراض المدارية المهملة هي مجموعه غير متجانسة من الامراض المزمنة والموهنة والتي غالبا ما تكون مرتبطة بالسكان ذوي الدخل المنخفض والمهمشين سياسيا، مما يسبب عبئا صحيا وإجتماعيا كبيرا في البلاد الموبوءة ويرجع السبب في هذه الأمراض للإصابة بمجموعه من مسببات الامراض بما في ذلك البكتيريا والطفيليات والكائنات الأولية والفيروسات ويعتبر التشخيص الدقيق للأمراض المدارية المهملة مهما للحد من نسبة الحالات المرضية ومنع الوفيات ورصد برامج المراقبة ويهدف تقييم الجودة الخارجية والذي يعتبر عنصرا من عناصر ضمان جوده المختبرات، إلى تقييم أداء المختبرات المشاركة في الكشف عن الإصابات الطفيلية والهدف من هذه الورقة البحثية هو وضع تقرير عن النتائج وطرح التوصيات المتعلقة ببناء القدرات من النتائج التي توصل اليها تقييم الجودة الخارجية لمختبرات الأمراض المدارية المهملة المشاركة في الدراسة في بلدان مختاره في منطقه منظمة الصحة العالمية غرب المحيط الهادئ من 2012 إلى 2015.

الأساليب: شاركت المختبرات المرجعية او مختبرات الصحة العامة العاملة في مجال الأمراض المدارية المهملة علي المستوي الوطني من 6 بلدان في تقييم الجودة الخارجية التي نظمه المعهد الوطني للأمراض الطفيلية التابع للمركز الصيني لمكافحه الامراض والوقاية منها والذي يقع بمدينة شنغهاي بالصين وتم دعوة ممثلين لكل مختبر للمعهد الوطني للأمراض الطفيلية للكشف عن إصابات الأمراض الطفيلية المدارية المهملة باستخدام نفس العينات المعدة للاختبارات المصلية التي تشمل مقايسة الممتز المناعي المرتبط بالإنزيم (إليزا) و التلاصق الدموي الغير مباشر والاختبارات المورفولوجية لبيض الديدان المعوية من خلال المسحة المباشرة وطريقة كاتو-كاتز وتم تسجيل جميع النتائج وتحليلها باستخدام النسخة رقم 19 من برنامج الحزمة الإحصائية للعلوم الاجتماعية.

النتائج: نسبة للمشاركين الذين حصلوا علي درجه تقييم الجودة الخارجية أعلي من او تساوي 60 خلال 2012-2015 لاختبار المسحة المباشر كانت 80 ٪ (2012) و71.43 ٪ (2013) و100 ٪ (2014) و75 ٪ (2015) في حين ان في حالة اختبار كاتو-كاتز كانت 80 ٪ (2012) و57.14 ٪ (2013) و100 ٪ (2014) و 37.5 ٪ (2015) علي التوالي وتفاوتت معدلات الكشف عن البيض في الأنواع المختلفة حيث كانت نسبتها مع الصفر الخراطيني (ديدان الأسكاريس) هي الأعلى بنسبة 94.07٪ في المتوسط وقد تفاعلت جميع المختبرات بشكل جيد للغاية مع إختبار مقايسة الممتز المناعي المرتبط بالإنزيم (إليزا) كما هو مبين بالدرجات العالية في جميع السنوات الأربع ما عدا مختبر أ في تقييم الجودة الخارجية الأول والأخير وللحكم بسلبية أو إيجابية عينات المصل فإن معدلات توافق الإليزا الإجمالية بين 2012 و 2015 كانت 90 ٪ و 99.29 ٪ و 94.29 ٪ و 98.75 ٪ علي التوالي بينما كانت معدلات التوافق الكلية للتلاصق الدموي الغير مباشر 100٪ و 95٪ و 90٪ و 97.5٪ علي التوالي ومع ذلك فان الكشف عن المستويات المنخفضة من الأجسام المضادة لمصل الدم لا يزال يمثل مشكله بالنسبة لطريقة التلاصق الدموي الغير مباشر عند أخذ معيار العينات في الاعتبار.

الإستنتاج: تبين هذه الدراسة ان نظام تقييم الجودة الخارجية كان مفيدا للمختبرات المشاركة ويحدد هذا البرنامج بعض أوجه القصور اللازمة للتغلب على مشاكل أداء المختبرات وتحسينها في تشخيص الديدان المعوية ومع ذلك لا يزال تحسين الدقة والتوحيد في تشخيص الأمراض المدارية المهملة يمثل تحديا كبيرا.

Translated from English version into Arabic by Mohamed R. Habib

从 2012-2015 年 WHO/西太区开展的实验室外部质量评估结果谈加强被忽视的热带病实验室检测能力的迫切性

卢艳，格伦达.冈萨雷斯，陈韶红，李浩，蔡玉春，储言红，艾琳，陈木新，陈海宁，陈家旭

摘要
引言：被忽视的热带病（Neglected tropical diseases, NTDs）是一类慢性的、致畸致残以及可造成生理缺陷使患者受歧视的疾病的总称，这类疾病主要影响贫穷及政治边缘化的人群，对流行区的公共卫生、社会经济造成严重负担。NTDs 涉及的病原体种类众多，包括细菌、寄生虫、螺旋体和病毒。NTDs 的确诊对降低发病率、减少死亡和监测疾病控制规划具有重要意义。外部质量评估是实验室质量保证体系的一部分，目的是评估参与实验室检测寄生虫感染的能力。本文报道了 2012-2015 年世界卫生组织西太区选定的几个国家 NTDs 实验室参与外部质量评估的结果以及在此基础上对实验室能力建设的建议。
方法：选择六个国家的 NTDs 参比实验室/公共卫生实验室参加由中国疾病预防控制中心寄生虫病预防控制所在上海组织的外部质量评估。每个实验室选派两名代表（作为一组）参与实验检测，分别用血清学方法（IHA 和 ELISA）、形态学方法（直接涂片法和改良加藤厚涂片法）检测鉴定相同样本。根据检测结果与样本阴阳性的一致性进行评分，并用 SPSS19.0 统计软件进行分析。
结果：形态学检测，直接涂片法中参与实验室得分≥60 的百分比分别为 80% (2012)、71.43% (2013 年)、100% (2014 年) 和 75% (2015 年)，而改良加藤厚涂片法得分≥60 的百分比分别为 80% (2012 年)、57.14% (2013 年)、100% (2014 年) 和 37.50% (2015 年)。蠕虫卵的检出率据种类不同有所差别；其中蛔虫卵的检出率最高，2012-2015 年平均为 94.07%。血清学方法，所有实验室在 ELISA 检测中均获得高分，仅 A 实验室 2012 年、2015 年的评估中得分较低。2012-2015 年对血清样本阴阳性的判断，ELISA 检测的总符合率分别为 90%、99.29%、94.29%和 98.75%，IHA 方法的总符合率则为 100%、95%、90%和 97.50%。IHA 检测中对于低水平血清抗体的滴度判定仍存在问题。
结论：结果表明通过外部质量评估可以发现实验室寄生虫病检测方面的不足，及需要着重加强的检测技能，同时也反映出 NTDs 诊断的准确性和相符性依然面临着挑战。

Translated from English version into Chinese by Yan Lu and Jia-Xu Chen

Les besoins urgents renforçant la capacité des laboratoires de Maladies Tropicales Négligées (MTNs) dans la Région du Pacifique Occidental de L’OMS: résultats de l'évaluation externe de la qualité sur le diagnostic des MTNs en 2012 – 2015

Yan Lu, Glenda Gonzales, Shao-Hong Chen, Hao Li, Yu-Chun Cai, Yan-Hong Chu, Lin Ai, Mu-Xin Chen, Hai-Ning Chen, Jia-Xu Chen

Résumé
Contexte: Les Maladies Tropicales Négligées (MTNs) constituent un groupe hétérogène de maladies principalement chroniques, débilitantes et souvent stigmatisantes qui affectent largement les populations à faible revenu et politiquement marginalisées, tout en causant un important fardeau de santé publique, sociale et économique dans les pays endémiques. Les MTNs sont causées par des infections dues à toute une gamme d'agents pathogènes, notamment des bactéries, des parasites, des protozoaires et des virus. Le diagnostic précis des MTNs est d’importance capitale afin de réduire la morbidité, prévenir la mortalité et de suivre les programmes de contrôle. L'Evaluation Externe de la Qualité (EEQ), une composante de l’assurance qualité des laboratoires, vise à évaluer le rendement des

laboratoires participants dans l'étude de détection d'infections parasitaires. Le but du présent document est de faire rapport sur les résultats et présenter les recommandations sur le renforcement des capacités à partir des résultats de l'EEQ des laboratoires participants dans les pays sélectionnés de la Région du Pacifique Occidental de L'OMS de 2012 à 2015.

Méthodes: Les laboratoires de référence de MTNs ou de santé publique au niveau national dans six pays ont participé aux EEQs organisées par l'Institut National des Maladies Parasitaires (NIPD), Centre Chinois de Contrôle et de Prévention des Maladies basé à Shanghai, Chine. Deux représentants de chaque laboratoire participant ont été invités à NIPD afinde mener des études détection d'infections parasitaires tropicales négligées en utilisant les mêmes échantillons préparés pour des tests sérologiques (IHA et ELISA) et des tests morphologiques des œufs d'helminthes (frottis et Kato-Katz). Tous les résultats ont été examinés et analysés à l'aide da la version 19.0 du logiciel SPSS de statistiques.

Résults: Le pourcentage de participants qui avaient un résultat d'EEQ $\geq$ 60 en 2012 - 2015 pour le test de frottis direct était de 80% (2012), 71,43% (2013), 100% (2014) et 75% (2015), tandis que le test Kato-Katz était de 80% (2012), 57,14% (2013), 100% (2014) et de 37,5% (2015), respectivement. Le taux de détection d'œufs d'helminthes varie selon les espèces, où A*scaris lumbricoides* étant le plus élevé, estimé à 94.07% en moyenne. Tous les laboratoires ont donné de très bonnes performances comme le témoigne le score élevé des tests ELISA durant les quatre ans, sauf le laboratoire A dans les première et dernière EEQs. Des résultas positifs ou négatifs obtenus à partir des échantillons de sérum, il en résulte que les taux de coïncidence totale pour le test ELISA entre 2012 et 2015 étaient de 90%, 99,29%, 94.29% et 98.75%, respectivement. Alors que les taux coïncidence totale du test IHA étaient respectivement 100%, 95%, 90% et 97,5%. Toutefois, la détection de faibles concentrations d'anticorps sériques demeure problématique pour le test IHA lorsque les titres des échantillons ont été pris en considération.

Conclusion: Cette étude démontre que le programme EEQ a été bénéfique pour les laboratoires participants. Le programme EEQ présente certaines lacunes qui auraient été nécessaires afin de surmonter et améliorer le rendement de diagnostic des helminthiases dans les laboratoires. Toutefois, davantage optimisation de l'exactitude et de l'uniformité du diagnostic des MTNs demeurent un grand défi.

Translated from English version into French by Kokouvi Kassegne

Срочно нуждается в укреплении забытых тропических болезней (NTDs) лабораторного потенциала в западной части Тихого океана ВОЗа: результаты внешней оценки качества дляNTDs диагностики в 2012-2015 годах

Yan Lu, Glenda Gonzales, Shao-Hong Chen, Hao Li, Yu-Chun Cai, Yan-Hong Chu, Lin Ai, Mu-Xin Chen, Hai-Ning Chen, Jia-Xu Chen

Реферат

Введение: Игнорируемые тропические болезни (NTDs) в основном представляют собой гетерогенную групп хронических, изнурительных и часто стигматизирующих заболеваний, что в значительной степени влияет на низкий доход и политически маргинализированные группы

населения, такой результат возникает большую нагрузку на здравоохранение, социальное и экономики в NTDs эндемичных странах. NTDs вызваны инфекциями ряд патогенов, включая бактерии, паразиты, простейшие и вирусы. Точный диагноз NTDs имеет важное значение для снижения заболеваемости, предотвращения смертности и контроля управляющих программ. Внешняя оценка качества (EQA), это компонент обеспечения качества лабораторных исследований, направленный на оценку эффективности участия лабораторий в выявлении паразитарных инфекций. Цель данной работы заключается в том, чтобы рассказывать выводе и выдвинуть рекомендации о возможности построитства из результатов EQA участвующих NTDs лабораторий в отдельных странах в Западно-Тихоокеанском регионе ВОЗ с 2012 по 2015 год.

Методы: Ссылка или лабораторий общественного здравоохранения на национальном уровне влияют на NTDs в 6 странах, которые участвовали в EQAs и были организованы Национальным Институтом паразитарных заболеваний (NIPD) китайского Центра по контролю и профилактике заболеваний (CDC), базирующегося в Шанхае, Китай. Два представителя из каждой лаборатории-участницы пригласили в NIPD для выявления паразитарных инфекций болезней с помощью той же подготовленных образцев для серологических тестов (IHA и ELISA) и морфологического анализа яйца helminth (Direct smear and Kato-Katz). Все результаты оценивались и анализировались с помощью статистических программ SPSS 19.0.

Результаты: Процент участников, у которых результат EQA ≥60 в течение 2012-2015 года для тиспытания прямого мазка были 80% (2012), 71.43% (2013), 100% (2014) и 75% (2015), в то время как для Kato-Katz испытания были 80% (2012), 57.14% (2013), 100% (2014) и 37,5% (2015), соответственно. Выявлене частоты яиц helminth отличается отдругих разных видов, Аскарида lumbricoides имеет самую высокую частату, это 94.07% в среднем. Все лаборатории очень хорошо разработали с анализами ELISA, как показанно высокими баллами во всех четырех лет, за исключением лаборатории в первом и последнем EQA. Для позитивных или негативных суждений образцов сыворотки крови, общая стоимость совпадения EQA в период между 2012 и 2015 годами были 90%, 99.29%, 94.29% и 98.75%, соответственно. В то время как общий коэффициент совпадения IHA составили соответственно 100%, 95%, 90% и 97,5%. Однако, обнаруживие низкие уровни сывороточных антител остаются проблематичным для IHA, когда титры образцов были принятыво внимание.

Заключение: Эти исследования демонстрируют, что схема EQA была выгодна для участвующих лабораторий. Программа EQA выявляет определенные недостатки, которым необходимо преодолеть и повысить производительность лабораторий в helminthiasis диагностике. Однако, дальнейшая оптимизация точности и единообразия в диагностике болезней остается большой проблемой.

Translated from English version into Russian by Hao-Qi Zhang

Necesidad urgente de fomentar la capacidad de laboratorio para las enfermedades tropicales desatendidas (ETDs) en la Región del Pacífico Occidental de la OMS: resultados de la evaluación externa de la calidad del diagnóstico de las ETDs en 2012-2015

Yan Lu, Glenda Gonzales, Shao-Hong Chen, Hao Li, Yu-Chun Cai, Yan-Hong Chu, Lin Ai, Mu-Xin Chen, Hai-Ning Chen, Jia-Xu Chen

Abstracto

Trasfondo: Las enfermedades tropicales desatendidas (ETDs) constituyen un grupo heterogéneo de enfermedades principalmente crónicas, debilitantes y, a menudo, estigmatizantes, que afectan en gran medida a las poblaciones de bajo ingreso y políticamente marginadas, causando una gran carga de salud pública, social y económica en los países endémicos de las ETDs. Las ETDs son causadas por infecciones por una variedad de patógenos, incluyendo bacterias, parásitos, protozoos y virus. El diagnóstico preciso de las ETDs es importante para reducir la morbilidad, prevenir la mortalidad y para supervisar los programas de control. Una evaluación externa de calidad (EEC), un componente de la garantía de la calidad de un laboratorio, tiene como objetivo evaluar el desempeño de los laboratorios participantes en la detección de infecciones parasitarias. El objetivo de este trabajo es informar de los hallazgos y presentar las recomendaciones sobre la creación de capacidad a partir de los resultados de la EEC de los laboratorios participantes de ETDs en países seleccionados de la Región del Pacífico Occidental de la OMS de 2012 a 2015.

Métodos: Los laboratorios de referencia o de salud pública que trabajan con enfermedades tropicales desatendidas a nivel nacional en 6 países participaron en las EEC organizadas por el Instituto Nacional de Enfermedades Parasitarias (NIPD, por sus siglas en inglés) del Centro de China para el Control y la Prevención de Enfermedades (CDC, por sus siglas en inglés). Dos representantes de cada laboratorio participante fueron invitados al NIPD para detectar las infecciones parasitarias de ETDs utilizando las mismas muestras preparadas para las pruebas serológicas (IHA y ELISA) y las pruebas morfológicas de huevos de helmintos (baciloscopía directa y Kato-Katz). Todos los resultados fueron puntuados y analizados mediante el uso del software *SPSS statistics 19.0*.

Resultados: El porcentaje de participantes que obtuvieron una puntuación de EEC ≥ 60 durante el período 2012-2015 para la prueba de frotis directo fue de 80% (2012), 71,43% (2013), 100% (2014) y 75% (2015), mientras que para Kato-Katz 80% (2012), 57,14% (2013), 100% (2014) y 37,5% (2015), respectivamente. La tasa de detección de huevos de helmintos varió en diferentes especies, siendo *Ascaris lumbricoides* el más alto con 94,07% en promedio. Todos los laboratorios hicieron muy bien las pruebas ELISA, como lo demuestran las puntuaciones altas en los cuatro años, excepto el laboratorio A en la primera y última EEC. Para los juicios positivos o negativos de las muestras de suero, las tasas de coincidencia total de ELISA entre 2012 y 2015 fueron de 90%, 99.29%, 94.29% y 98.75%, respectivamente. Mientras que las tasas de coincidencia total de IHA fueron respectivamente 100%, 95%, 90% y 97,5%. Sin embargo, la detección de niveles bajos de anticuerpos séricos siguió siendo problemática para IHA cuando se tomaron en consideración los títulos de las muestras.

Conclusión: Este estudio demuestra que el esquema EEC ha sido beneficioso para los laboratorios participantes. El programa EEC identificó ciertas deficiencias que eran necesarias superar para mejorar el desempeño de los laboratorios en el diagnóstico de helmintiasis. Sin embargo, una mayor optimización de la exactitud y la uniformidad en el diagnóstico de las ETDs sigue siendo un gran desafío.

Translated from English version into Spanish by Laura C Vicente Rodriguez
